# The Hemodialysis Distress Thermometer for Caregivers (HD-DT-C): development and testing of the psychometric properties of a new tool for screening psychological distress among family caregivers of adults on hemodialysis

**DOI:** 10.1007/s11136-024-03627-x

**Published:** 2024-03-07

**Authors:** Helena Sousa, Oscar Ribeiro, Daniela Figueiredo

**Affiliations:** 1 CINTESIS@RISE, Department of Education and Psychology, University of Aveiro - Campus Universitário de Santiago, Edifício 5, 3810-193 Aveiro, Portugal; 2 CINTESIS@RISE, School of Health Sciences, University of Aveiro, Aveiro, Portugal

**Keywords:** Kidney failure, Dialysis, Distress, Quality of life, Caregiver, Health-related outcome measure

## Abstract

**Purpose:**

To develop and test the measurement properties of the HD-DT-C, a new tool designed to facilitate the screening of psychological distress and its sources in family caregivers of adults on hemodialysis.

**Methods:**

The present investigation was carried out in three phases: Phase 1 focused on the process of developing and exploring the content validity and clinical utility of the HD-DT-C using a mixed-methods approach and feedback panels; Phase 2, where the psychometric properties of this new tool were tested in a cross-sectional study (*n* = 106 caregivers); and Phase 3, where the European Portuguese version of the HD-DT-C was translated and culturally adapted into American English using a forward–backward translation procedure, followed by an expert panel review.

**Results:**

Findings suggested that the HD-DT-C was perceived by feedback panels as practical, appropriate, and useful for increasing dialysis provider/family caregiver communication in nephrology centers. The European Portuguese version of the HD-DT-C showed good test–retest reliability (*ICC* = 0.991 for the barometer and *κ* ≥ 0.80 in 77% of the checklist items), high diagnostic accuracy (*AUC* = 0.956), and strong convergent validity (all *r* ≥ 0.50) with reference measures that assess quality of life, caregiver burden, and symptoms of anxiety and depression. Cutoff scores with good clinical utility (*CUI* +  ≥ 0.70) were recommended for screening distress in research (≥ 6) and clinical practice (≥ 5).

**Conclusion:**

The HD-DT-C is a brief, reliable, valid, and acceptable measure for identifying self-reported psychological distress and its sources among people caring for a family member or friend on hemodialysis. Future research is needed to explore the measurement properties of the American English version of this new tool.

**Supplementary Information:**

The online version contains supplementary material available at 10.1007/s11136-024-03627-x.

## Introduction

Worldwide, hemodialysis is the most common renal replacement therapy for adults with kidney failure [1]. This life-sustaining treatment is extremely demanding and often associated with significant impairments in the physical and emotional functioning of those living with this serious health condition, increasing their dependence on close family members [2]. Providing care and assistance to a person receiving renal therapy can be an arduous experience, involving feelings of burden and a constant need to readjust personal, family, and social activities and life goals to meet dialysis caregiving demands [3]. In this regard, research has consistently shown that psychological distress is prominent among hemodialysis caregivers, with approximately 25% of this population reporting symptoms of depression, sleep problems, and poor quality of life [3, 4]. Studies have also stated that the levels of psychological distress perceived by family caregivers of adults on hemodialysis are higher than those reported by the person being cared for [5], and/or close relatives of individuals with other serious health conditions such as cancer [6]. If not promptly addressed, caregiver distress can progress to mood and anxiety disorders [7, 8] which, in turn, have been associated with worse health outcomes for care recipients (e.g., low treatment adherence, recurrent hospitalizations) and an increase in healthcare service utilization and costs [9]. Despite this growing awareness, the well-being of family caregivers of people with kidney failure remains overlooked and under-prioritized in most renal care settings around the world [10, 11].

In clinical contexts such as oncology or dementia care, screening caregivers' psychological symptoms has proven to be a promising approach for improving their integration into educational and/or support intervention programs [12–14]. However, the lack of measures that allow the rapid identification of psychological distress and the early detection of the unique difficulties faced by people supporting a family member or friend undergoing renal therapy continues to be an important barrier to the organization of psychosocial initiatives (screenings and/or interventions) in nephrology [15, 16]. Given the paucity of tools specifically designed for this population, research has frequently resorted to measures that assess the presence of symptoms of anxiety, depression, and burden as constructs related to psychological distress (e.g., the Hospital Anxiety and Depression Scale [HADS], Beck Depression Inventory [BDI], Beck Anxiety Inventory [BAI], Zarit Burden Interview [ZBI]) [4, 17]. However, although some of these tools have been validated and established for use in non-clinical samples, their psychometric evidence remains poorly explored in hemodialysis caregivers [16]. Moreover, these measures do not address the complex and unique demands of providing informal care in the context of this renal replacement therapy, which may therefore reduce their clinical utility [16, 18].

To date, and to the best of our knowledge, there are no tools specifically designed for screening psychological distress in family caregivers of adults on hemodialysis. The development of easy-to-administer, reliable, and valid measures for this purpose is therefore of utmost importance for research and clinical practice in nephrology care, as it may represent a crucial step in facilitating early detection and monitoring of the difficulties faced by these caregivers.

The present study aimed to develop, evaluate the clinical utility, and test the measurement properties of the Hemodialysis Distress Thermometer for Caregivers (HD-DT-C), a new tool for screening, identifying, and monitoring self-reported psychological distress and its sources among family caregivers of adults undergoing hemodialysis. This measure is also intended to assist in the recruitment of caregivers for observational and interventional studies (e.g., using distress levels as an eligibility criterion), and/or help inform the design and evaluation of the effectiveness of self-management interventions aimed at this population.

## Methods

### Study design

**Fig. 1:**
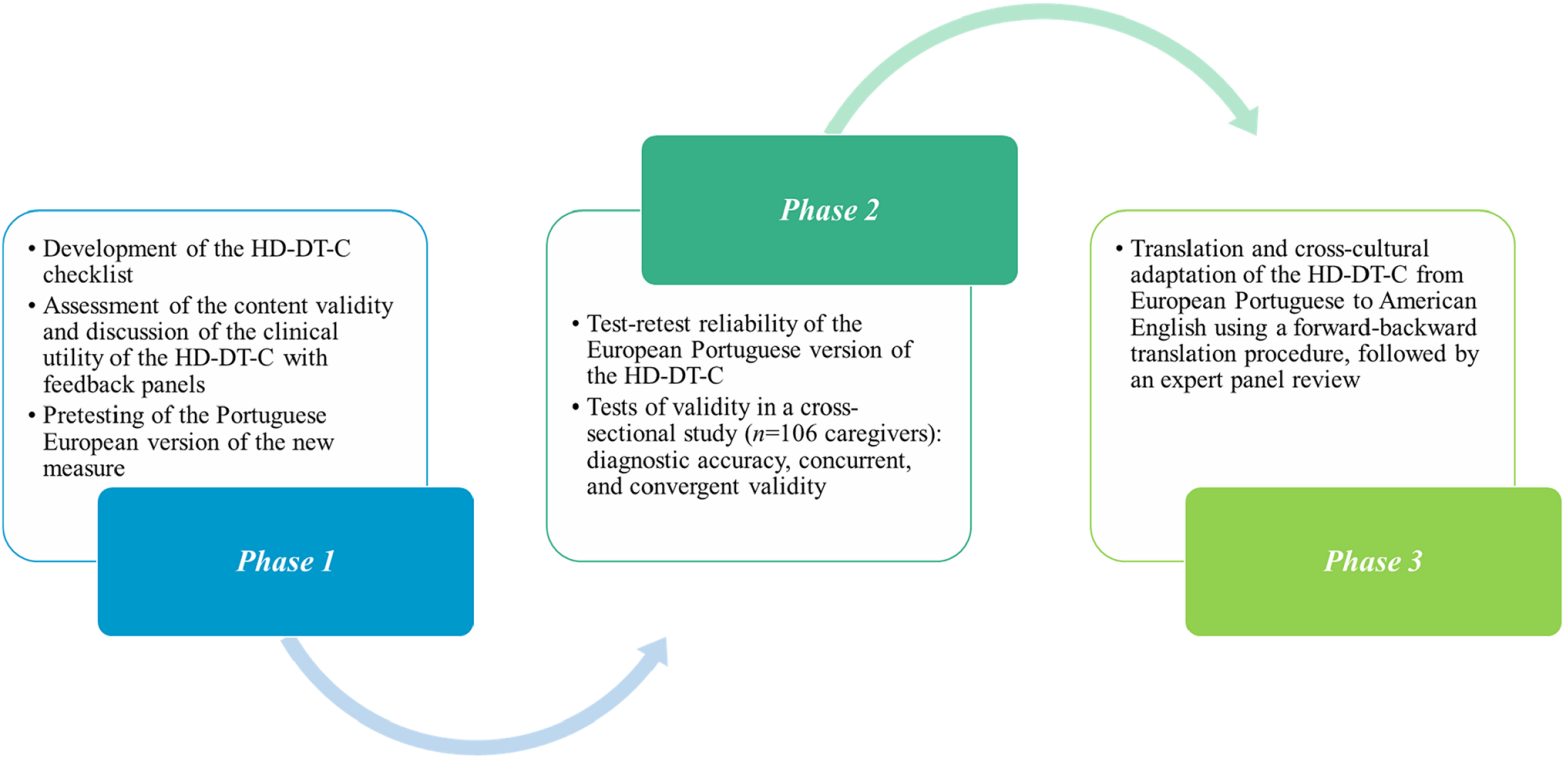
The process of developing a new tool for screening psychological distress and its sources among family caregivers of adults on hemodialysis: the Hemodialysis Distress Thermometer for Caregivers (HD-DT-C)

The current investigation was conducted in three consecutive phases (Fig. 1): Phase 1 focused on the process of developing and evaluating the content validity and clinical utility of the HD-DT-C using a mixed-methods approach and two feedback panels; in Phase 2, the goal was to test the psychometric properties of the European Portuguese version of the HD-DT-C in a cross-sectional descriptive-correlational study; and, in Phase 3, the HD-DT-C was translated and culturally adapted into American English using a forward–backward translation procedure and review by a panel of experts.

### Phase 1: process of developing and evaluating the content validity and clinical utility of the HD-DT-C

The HD-DT-C was designed to consist of a barometer with a single-item question ranging from 0 (no distress) to 10 (extreme distress) and a checklist of caregiving-related stressors frequently reported by hemodialysis caregivers. This framework was defined a priori as it has been widely and successfully used in other measures that aim to identify distress and its sources among family caregivers (and patients) in different healthcare contexts (e.g., cancer, inflammatory bowel diseases, multiple sclerosis) [17]. An example of a tool that follows this alignment is the National Comprehensive Cancer Network Distress Thermometer (NCCN-DT), a measure that has been internationally validated to screen distress in oncology centers [19].

The HD-DT-C checklist items were generated by combining the results of a literature search of instruments specifically designed to assess hemodialysis-related caregiving stressors, the examination of items from distress thermometers validated in other caregiving contexts (e.g., oncology care), and a secondary thematic analysis of the transcribed interviews of family caregivers of adults undergoing hemodialysis (*n* = 32) and dialysis care professionals (*n* = 23). The literature search was performed between January and June 2022, on Web of Science Core Collection (all databases included), MEDLINE/PubMed, and CINHAL. The following keywords were used: ‘dialysis’, ‘hemodialysis’, ‘PROM’, ‘measure’, ‘scale’, ‘questionnaire’, ‘instrument’, ‘tool’, ‘caregiver’, ‘relative’, ‘family’, and ‘spouse’, and only studies written in English, Spanish, French, and Portuguese were considered. In turn, the qualitative interviews were analyzed following Braun and Clark's [20] recommendations for data categorization. These data were derived from a larger research project that aimed to deepen the understanding of the impacts of kidney failure from the perspective of the triad people on hemodialysis/family caregivers/dialysis care professionals. Sample recruitment and procedures for data collection and analysis were published elsewhere [21].

A mixed-methods approach (calculation of the content validity index and findings from focus group interviews) was then adopted to assess the content validity of the HD-DT-C using two independent feedback panels: a panel of dialysis experts (*n* = 9) and a group made up of judges from the target population (*n* = 10 caregivers). The results of the Item-Content Validity Index (I-CVI) [22] informed the decision to review and/or exclude items from the tool under development. The potential clinical utility of HD-DT-C (and how to maximize it) in renal care settings was also discussed during focus group interviews, guided by the theoretical foundations of Smart's multidimensional model of clinical utility [18] (cf. Online Resource 1).

Before testing its psychometric properties, the final version of the HD-DT-C was pretested with a convenience sample of family caregivers (*n* = 11) recruited from two dialysis centers where the cared-for person with kidney failure was receiving treatment. During pretest, these participants completed the HD-DT-C and an Acceptability Questionnaire that aimed to evaluate their opinions about the new measure (e.g., understanding of the items; level of effort, and perceived difficulty in completing the measure; suitability, usefulness, practicality, and potential acceptability of the HD-DT-C if administered regularly) (cf. Online Resource 2).

The stepwise approach that informed the development of HD-DT-C is presented in Table 1. The patient version (HD-DT) can be found elsewhere along with a more detailed description of this stepwise approach [manuscript submitted to another journal at the time of submission of the current work]. Procedures for item generation and assessment of content validity of both measures followed the recommendations of Boateng et al. [23] to design health, social, and behavioral measures, and the COnsensus‐based Standards for the selection of health Measurement INstruments (COSMIN) guidelines [24].

**Table 1:** The eight steps that informed the development of the HD-DT-C

STEP*	Definition	Description
STEP 1	Content and domain specification	The following definitions were adopted to bind the content and domains assessed by the HD-DT-C: • *Psychological distress:* state of emotional suffering resulting from the caregivers’ perception of the depletion of coping resources to deal with a threatening situation [8]. It can encompass (but is not limited to) symptoms of anxiety, depression, stress, and burden that, if left untreated, can progress to psychopathological disorders [7, 8] • *Stressor*: any event, force, or condition that results in physical or emotional stress and requires adjustments or coping strategies on the part of the affected caregiver [62]
STEP 2	Checklist item generation	The HD-DT-C checklist items were generated by combining deductive and inductive methods deriving from three different sources (literature search, items from other distress thermometers, and qualitative interviews with family caregivers of people on hemodialysis and dialysis care professionals)
STEP 3	Preliminary item selection	Stressors retrieved from Step 2 were checked to find overlaps. Those deemed to be significantly overlapping were combined into a single item (e.g., the stressors ‘having all the responsibility for caring’, ‘lack of support from family members in caregiving’, ‘other family members do not accompany me in caring’, ‘other family members and friends don’t help me move my patient and bring him to the hospital’, and ‘I do not feel the sympathy and support of my loved ones’ were merged into one single item: ‘lack of family support in the distribution of caregiving responsibilities’)
STEP 4	Item clustering	Preliminary items were grouped according to their content similarity to generate the sections of the HD-DT-C checklist
STEP 5	Content validity assessment	Based on the proportion of items classified by experts/judges as relevant (i.e., ≥ 3 on a Likert scale ranging from 1 = 'not relevant' to 4 = 'highly relevant'), three content validity indexes were calculated: experts I-CVI, caregivers I-CVI, and total I-CVI. Items were discussed during focus group interviews if at least one of the I-CVI indicators was ≥ 0.70; items with all I-CVI indicators < 0.70 were considered irrelevant and therefore eliminated [22]
STEP 6	Evaluation of the clinical utility of the new measure	The potential clinical utility of HD-DT-C in renal care settings was discussed with feedback panels during focus group interviews conducted by a health psychologist with experience in this type of research
STEP 7	Final item selection	The data obtained in these previous steps were analyzed and discussed by the research team during several sequential group meetings that led to the final version of the HD-DT-C
STEP 8	Pretesting	The final version of the HD-DT-C was pretested using LimeSurvey, a secure online platform for administering the questionnaires with implied consent (http://www.limesurvey.org). The time required to complete the HD-DT-C was recorded using this platform. Respondents were also invited to answer an acceptability questionnaire developed by the research team to assess the acceptability of the HD-DT-C and the HD-DT (the patient version) (available for consultation in Online Resource 2)

*The development of the HD-DT-C was also based on a formative (vs. reflective) model, meaning that each stressor/checklist item is viewed as causing psychological distress (the latent variable) [63]

### Phase 2: testing the psychometric properties of the European Portuguese version of the HD-DT-C

The recommendations of the COSMIN taxonomy [25] were followed to inform the test of the measurement properties of the HD-DT-C.

#### Test–retest reliability

Caregivers who participated in the HD-DT-C pretest (*n* = 11) were invited to repeat the measure 7 to 10 days later. For data analysis, intra-rater reliability with a 95% confidence level was obtained by computing the Intraclass Correlation Coefficient (*ICC*) for the HD-DT-C barometer based on a mean-rating (*k* = 2), two-way mixed effects analysis of the variance model with interaction for the absolute agreement. Cohen's Kappa was calculated for each of the categorical items in the checklist. All statistics were computed using IBM SPSS (Version 28.0). Statistical significance was set as *p* < 0.05.

#### Tests of validity

The concurrent and convergent validity of the European Portuguese version HD-DT-C were tested against predetermined reference standards (Table 2).

**Table 2:** Procedures adopted to validate the European Portuguese version of the HD-DT-C

Reference standard	Description	Tests of validity [statistical analysis]
Hospital Anxiety and Depression Scale (HADS)	14-item scale that assesses the presence of symptoms of anxiety (HADS-A) and depression (HADS-D) [27]. The HADS total score (HADS-T) is considered a global measure of psychological distress and has been the most frequently used criterion measure in validation studies of distress barometers [17, 18]. To the best of our knowledge, there are no established cutoff values ​​for the use of the HADS-T in Portuguese samples [28]; therefore, a cutoff point ≥ 15 was adopted to determine the presence of clinically relevant distress in the current investigation. This methodological choice was informed by the recommendations of a recently published meta-analytical study that explored the psychometric properties of the HADS-T in 98 primary studies with clinical and non-clinical populations [64]. The HADS showed good internal consistency for the present study sample with all Cronbach's alphas > 0.80 (HADS-A: *α* = 0.834; HADS-D: *α* = 0.851; HADS-T: *α* = 0.911)	Diagnostic accuracy and concurrent validity of the HD-DT-C barometer against the HADS-T [ROC analysis] Convergent construct validity of the HD-DT-C (barometer and checklist^a^) against the HADS-A and HADS-D [Pearson’s *r*] *Hypothesis tested for construct validity:* The results on the HD-DT-C (barometer and checklist^a^) have a strong positive correlation (*r* ≥ 0.50) with scores on the HADS-A and HADS-D
World Health Organization's Quality of Life Instruments – BREF (WHOQ-BREF)	26-item instrument that measures the individual's perception of ‘overall quality of life’ and ‘general health’ (2 items), ‘physical health’ (7 items), ‘psychological health’ (6 items), ‘social relationships’ (3 items), and ‘environmental health’ (8 items). This tool has been administered in a wide variety of settings, including with family caregivers of people with kidney failure [65]. The validity of the HD-DT-C was tested against the ‘psychological health domain’ of the WHOQOL-BREF. The Portuguese version of this instrument [29] showed good internal consistency for the present study sample (*α* = 0.785 in the 'psychological health domain')	Convergent construct validity of the HD-DT-C (barometer and checklist^a^) against the ‘psychological health domain’ of the WHOQOL-BREF [Pearson's *r*] *Hypothesis tested for construct validity:* The results on the HD-DT-C have a strong negative correlation (*r* ≥ 0.50) with scores on the ‘psychological health domain’ of WHOQOL-BREF
Zarit Burden Interview (ZBI)	22-item questionnaire that is considered a reference measure to assess caregiver burden [30]. This tool has been widely used in studies with family caregivers to explore the self-perceived burden of supporting a person with kidney failure [4]. The Portuguese version of the ZBI [31] showed good internal consistency for the present study sample (*α* = 0.940 for the total score)	Convergent construct validity of the HD-DT-C (barometer and checklist^a^) against the ZBI total score [Pearson's *r*] *Hypothesis tested for construct validity:* The results on the HD-DT-C have a strong positive correlation (*r* ≥ 0.50) with the ZBI total score

*HD-DT-C* Hemodialysis Distress Thermometer for Caregivers, *HADS* Hospital Anxiety and Depression Scale, *WHOQ-BREF* World Health Organization Quality of Life Instruments–BREF, *ZBI* Zarit Burden Interview

^a^Validity tests of the HD-DT-C checklist (categorical variables) were performed for the total number of difficulties and/or concerns (numerical variable) pointed out by respondents

*Sample recruitment* Family caregivers of adults (≥ 18 years old) undergoing hemodialysis for a minimum of 2 months were eligible to participate. For the purpose of the current investigation, a family caregiver was defined as the family member (relative or partner) or friend who provided the most physical care and/or support to the person on hemodialysis without receiving any remuneration [4]. Participants were recruited from four dialysis units in Portugal, where the cared-for person was receiving renal therapy (convenience sample). To this end, people with kidney failure were asked to indicate their caregiver and authorize the researchers’ contact to assess their availability and potential interest in joining the present investigation. Potential respondents under 18 years old were not considered; only those who did not have any visual, auditory, and/or neurocognitive impairment that could hinder understanding of the purpose of the study were considered.

A sample size of 114 respondents was estimated to test the concurrent validity and diagnostic accuracy of the HD-DT-C and achieve a predetermined moderate accuracy value with an Area Under the Curve (AUC) ≥ 0.70 with a 95% confidence interval with an estimation accuracy of 0.07 [26].

*Data collection* Participants completed a set of questionnaires over the phone or using a web-based survey displayed on LimeSurvey, according to their preference and/or convenience. The psychometric assessment protocol included a questionnaire collecting sociodemographic and clinical information, the HD-DT-C, and other self-report measures. The Hospital Anxiety and Depression Scale (HADS) [27, 28], World Health Organization's Quality of Life Instruments—BREF (WHOQ-BREF) [29], and Zarit Burden Interview (ZBI) [30, 31] were used as reference standards (Table 2). Data collection took place between September 2022 and February 2023.

*Data analysis* Receiver operating characteristic (ROC) analysis was used to compute the AUC and the sensitivity and specificity of the HD-DT-C barometer against the total HADS score (HADS-T). Cutoff scores were determined by finding the point that maximizes sensitivity and specificity [32]. To confirm the accuracy of the optimal cutoff, the Clinical Utility Index (*CUI* +) [33] and the Youden Index (*J*) [34] were estimated. The convergent validity of the HD-DT-C was obtained using Pearson’s product-moment correlation coefficient (*r*).

### Phase 3: translation and cross-cultural adaptation of the HD-DT-C into American English

The HD-DT-C was translated and culturally adapted simultaneously with the patient version (HD-DT) and following the recommendations of Beaton et al. [35]. Overall, the HD-DT-C was translated from European Portuguese to American English by a trained and certified translator, while back-translation was performed by a bilingual, independent researcher with experience in psychometrics. After the reconciliation of both versions, an expert panel (*n* = 6) rated the semantic, idiomatic, and cultural adequacy of the translated version of the tool. Three of these experts were fluent in both the source and target language, while the other three were monolingual native speakers of the target language. Afterward, the research team made the necessary reconciliations and achieved a final translation of the measure. The necessary changes were made to culturally adapt the translated version of the HD-DT-C to try to maximize the cultural equivalence of the measure to the American context.

The American English version of the HD-DT-C is available for consultation in Online Resource 3. Future studies are needed to pretest this translated version and test its psychometric properties in U.S. samples.

## Results

### Phase 1 results

#### Development of the HD-DT-C checklist

The literature search identified three available health-related outcome measures specifically designed for hemodialysis caregivers, namely the Renal Caregiver Burden Scale [36], the Perceived Care Tension Questionnaire for Caregivers of Hemodialysis Patients [37], and the Health-Related Quality of Life Inventory for Family Caregivers of Hemodialysis Patients [38]. The examination of items from these tools resulted in an initial set of 98 caregiving stressors. Thirty-eight items were also retrieved from distress thermometers validated for use with caregivers of people with other chronic health conditions (e.g., the NCCN DT) [17, 18].

A secondary thematic analysis of the transcribed interviews of family caregivers (*n* = 32) and dialysis care professionals (*n* = 23) was performed to explore their perspectives on hemodialysis-related caregiving stressors. Participants in the caregiver group had a mean age of 51.9 years (*SD* = 14.7; *min* = 21; *max* = 80); the majority were adult children (50%) or spouses (44%) of people with kidney failure for whom they had been caring for more than four years (34%). In turn, renal nurses and nephrologists had a mean age of 38.1 years (*SD* = 10.1; *min* = 26; *max* = 64) and had been working in nephrology care for an average of 11 years (*SD* = 9.63; *min* = 3; *max* = 36).

Thirteen stressors were identified from the interviews with family caregivers: (1) needing more information about what is recommended for the diet of an adult on hemodialysis; (2) negating certain foods/meals to the family member with kidney failure; (3) being creative with meals so that the cared-for person does not lose appetite; (4) denying fluids when the person undergoing renal therapy feels thirsty; (5) not knowing the recommended amount of fluids for an adult on hemodialysis to ingest daily; (6) difficulty managing the administration (and dosages) of the different medications that the cared-for person needs to take; (7) doubts on how to help the family member with the vascular access hygiene, hydration, and care; (8) handling fistula-related complications such as bleeding and bruising; (9) lack of support from close relatives with whom caregivers wished to share caregiving responsibilities; (10) managing strained relationships with family members who act as secondary caregivers; (11) not knowing how to cope with the negative feelings and reactions of the person being cared for toward kidney failure and renal therapies (e.g., distress, anger, resistance to treatment, thoughts on dialysis withdrawal); (12) managing negative feelings toward the cared-for person disease and/or treatments (e.g., sadness, frustration, hopelessness); and (13) feeling guilty about being less available to other family members, such as spouse and/or young children, due to caregiving responsibilities.

Regarding the perspectives of dialysis care professionals on the main stressors faced by people assisting a family member or friend with kidney failure, overlapping themes were found with the qualitative data obtained in the semi-structured interviews with family caregivers (concerns about fistula-related complications; struggling to emotionally support a close relative on dialysis; difficulties in dealing with the negative feelings of the person being cared for; managing nutritional recommendations, fluid restrictions, and polypharmacy prescriptions). Thus, seven new stressors emerged from the secondary thematic analysis of the transcribed interviews of renal nurses and nephrologists: (1) caregivers' difficulties in coping with the progressive decline in the physical and cognitive function of the family member with kidney failure; (2) dealing with the impacts of caregiving on life (personal, family, and social) goals and projects; (3) handling tense relationships with friends and co-workers; (4) loneliness; (5) feeling overwhelmed by caregiving responsibilities; (6) concerns about the kidney transplant of the person on dialysis (e.g., not finding a compatible donor, little information about kidney transplantation, unknown and/or prolonged waiting list for organ transplantation); and (7) worrying about the future health of the care recipient.

After combining the findings from the different sources and eliminating duplicates, a list of 150 hemodialysis-related caregiving stressors was generated (cf. Online Resource 4).

#### Item clustering

From this set of 150 stressors, those that were considered overlapping were combined into a single item (cf. example described in Table 1), resulting in a preliminary selection of 48 items. These were then grouped into the following clusters: physical stressors (*n* = 6 items), psychological stressors (*n* = 18 items), social/family stressors (*n* = 6 items), and stressors related to caregiving tasks (*n* = 18 items). Each of these clusters represents a section of the HD-DT-C checklist (cf. Online Resource 5).

#### Content validity assessment

The content validity of the HD-DT-C with a 48-item checklist was appraised by two independent feedback panels using a mixed methods approach. The expert panel (*n* = 9) was composed of seven renal nurses and two nephrologists from two different dialysis units, with a mean age of 35.9 years (*SD* = 8.77; *min* = 28; *max* = 67), who had been working in nephrology care for an average of 7.67 years (*SD* = 7.29; *min* = 3; *max* = 40). In turn, the target population group consisted of 10 caregivers (all women; six spouses, and four adult children of people with kidney failure), with a mean age of 51 years (*SD* = 7.01; *min* = 42; *max* = 62); these participants had been caring for a person on hemodialysis for an average of 62.1 months (*SD* = 27.7; *min* = 22; *max* = 120).

All I-CVI results for the relevance of each of the 48 items listed in the first draft of the HD-DT-C checklist are shown in Online Resource 5. Twenty-two (46%) items were rated as relevant (total I-CVI > 0.79), eight (17%) as not relevant (all I-CVIs < 0.70), while the remaining 18 (38%) were considered 'marginal' (at least one of the I-CVIs ≥ 0.70). Items classified as ‘not relevant’ were directly removed from the checklist, while ‘marginal’ items were discussed in the focus group interviews with both feedback panels, resulting in the elimination of 11 items that were deemed not relevant or better represented by another item already existing in the HD-DT-C. Some of the adjustments made to the HD-DT-C during focus group interviews with feedback panels (cf. Online Resource 6) followed the changes made to the patient version (HD-DT), as both measures were developed simultaneously with the same group of experts (e.g., translation of the Anglo-Saxon terms ‘distress’ and ‘stressor’, the definition of a recall period, the inclusion of two open questions). The name of the tool was set as ‘Hemodialysis Distress Thermometer for Caregivers’ in English and *‘Termómetro do Sofrimento Emocional da Hemodiálise para Cuidadores’* in Portuguese, with the acronym 'HD-DT-C' being adopted for both versions.

Additionally, two open questions were added to the measure under development after the focus group interviews with renal nurses, nephrologists, and family caregivers. One of these questions was intended to encourage respondents to describe other sources of distress while the other question aimed to inquire caregivers about their desire to receive support to cope with the sources of distress identified in the HD-DT-C.

#### Clinical utility of the HD-DT-C in renal care settings

The focus group interviews also allowed for discussion of the potential clinical utility of the HD-DT-C in nephrology centers, and the direct and indirect advantages and drawbacks of using this measure. Overall, findings suggested that both feedback panels perceived the HD-DT-C as useful, appropriate, and practical for screening psychological distress in caregivers of adults with kidney failure, *“since it is easy to fill out and you don't have to think a lot about each item and what it means. (…) It's simple and doesn't take long”* [caregiver].

In addition, experts/judges advocated the routine use of the HD-DT-C as a means of facilitating the communication between dialysis providers and caregivers and increasing the attention given to the (often unmet) needs of people supporting a family member or friend receiving renal therapy: “*We* [nurses] *rarely talk to caregivers and their needs are not very clear to us. (…) The use of this questionnaire can help to recover this communication that was lost over time*” [renal nurse]; “*I think this* [distress screening] *can help to give 'voice to the caregiver' because we often feel alone and left out. This is a way for them* [dialysis providers] *to know what worries us and find ways to help us”* [caregiver]. In this sense, the experts/judges recommended that the measure be completed by the caregiver when the person with kidney failure initiates dialysis (*“The family also needs a lot of support at this stage*” [renal nurse]) and then, periodically, at least once a year, since “*more than that can be upsetting for us”* [caregiver]).

Considering this, different options were explored to increase the accessibility of the HD-DT-C in renal care settings. There was consensus on the suggestion that the measure could be made available to caregivers by dialysis centers, upon invitation to fill out a questionnaire. The possibility of completing the HD-DT-C by telephone was indicated as an alternative for those who are unable to travel to the dialysis unit: “*My suggestion is to ask caregivers if they can come to the dialysis unit to fill out a questionnaire. (…) If the family member is not available to do so, they can fill out the questionnaire by telephone or it can also be sent by mail or through the patient”* [caregiver]. Renal nurses and/or social workers were proposed by feedback panels as the main agents of dissemination of the HD-DT-C since the presence of mental health professionals in nephrology teams is still scarce.

#### Final item selection and pretesting

These previous steps led to the final version of the HD-DT-C with a 30-item checklist that was then pretested with 11 caregivers (*M*_age_ = 50.7, *SD* = 10.3, *min* = 41, *max* = 67; 10 women; six spouses, and five adult children of people with kidney failure). These participants needed an average of 8.61 min (*SD* = 2.02; *min* = 4.27; *max* = 11.3) to fully complete the measure (barometer and checklist). The results of the ‘HD-DT-C Acceptability Questionnaire' showed that all caregivers (*n* = 11; 100%) recognized that they made little or no effort to fill out the HD-DT-C and evaluated the time needed to complete the instrument as being very acceptable. In addition, the degree of difficulty in classifying their level of distress on the barometer was rated as easy (*n* = 6; 55%) or very easy (*n* = 5; 45%), while the checklist items were considered very easy to understand by most respondents (*n* = 8; 73%). The HD-DT-C was also appraised as very adequate, useful, and practical (*n* = 11; 100%, respectively). All family caregivers who participated in the pretest of the European Portuguese version of the HD-DT-C stated that they would be willing to retake the measure at least once a year if suggested by the dialysis center where the person being cared for was undergoing treatment.

## Phase 2 results

### Test–retest reliability results

Online Resource 7 displays all the HD-DT-C test–retest reliability results. The ICC for the distress barometer showed excellent absolute agreement (*ICC* = 0.991, 95% CI 0.59–0.998, *p* < 0.001) among participant responses with an interval of 7 to 10 days between the pre- and retest assessment (*n* = 10 respondents; one of the caregivers who participated in the pretest was excluded from the retest evaluation, as the cared-for person was hospitalized during this period). Cohen's Kappas for the categorical items ‘caring for my family member affects my social and/or family life' (*κ* = 0.375, 95% CI − 0.329–1.079, *p* = 0.236) and 'changes in physical capacity' (*κ* = 0.412, 95% CI − 0.178–1.002, *p* = 0.107) had the lowest agreement rates. The results for the other items in the HD-DT-C checklist ranged from 0.615 to 0.8 (substantial agreement), with 77% (*n* = 23) of the items presenting Kappa values ≥ 0.8 which indicates almost perfect agreement between test and retest scores [39, 40].

#### Validity results

For the European Portuguese version of the HD-DT-C, concurrent and convergent validity was tested with a sample of 106 caregivers. Table 3 exhibits the sociodemographic and clinical characteristics of this sample.

**Table 3:** Characterization of the sample used to validate the European-Portuguese version of the HD-DT-C (*n* = 106)

Sample characteristics	Family caregivers (*n* = 106)
Women, *n* (%)	85 (80.2%)
Age (years old), *M* ± *SD* [range]	52 ± 16.1 [19–85]
*Educational level, n (%)*	
Primary education (4th grade)	27 (25.5%)
Basic education (6th grade)	15 (14.2%)
Basic education (9th grade)	19 (17.9%)
Secondary education (12th grade)	36 (34%)
Superior education	9 (8.5%)
*Kinship with the person with kidney failure, n (%)*	
Spouse (legally or otherwise)	46 (43.4%)
Other (parents, siblings, and adult children)	60 (56.6%)
*Length of time as a caregiver (in years), n (%)*	
< 2	47 (44.3%)
> 2	59 (55.7%)
Receiving professional psychological support, *n* (%)	4 (3.8%)
HADS-Depression subscale,* M* ± *SD* [range]	4.92 ± 4.18 [0–19]
HADS-Anxiety subscale,* M* ± *SD* [range]	7.85 ± 4.72 [0–18]
HADS-Total distress,* M* ± *SD* [range]	12.8 ± 8.46 [0–36]
WHOQ-BREF Psychological health domain, *M* ± *SD* [range]	70.7 ± 13.5 [21–96]
ZBI Total score, *M* ± *SD* [range]	41.7 ± 16.7 [22–87]

*HADS* Hospital Anxiety and Depression Scale (total score ranges from 0 to 42 for HADS-Total distress, and from 0 to 21 for the anxiety and depression subscales, respectively; higher scores indicate greater symptoms), *WHOQ-BREF* World Health Organization Quality of Life Instruments – BREF (total scores range from 0 to 100; higher values ​​correspond to a perception of a better quality of life in the ‘psychological health domain’ used in the validation study of the HD-DT-C), *ZBI* Zarit Burden Interview (total scores range from 22 to 110 with higher scores corresponding to higher levels of caregiver burden)

**Fig. 2:**
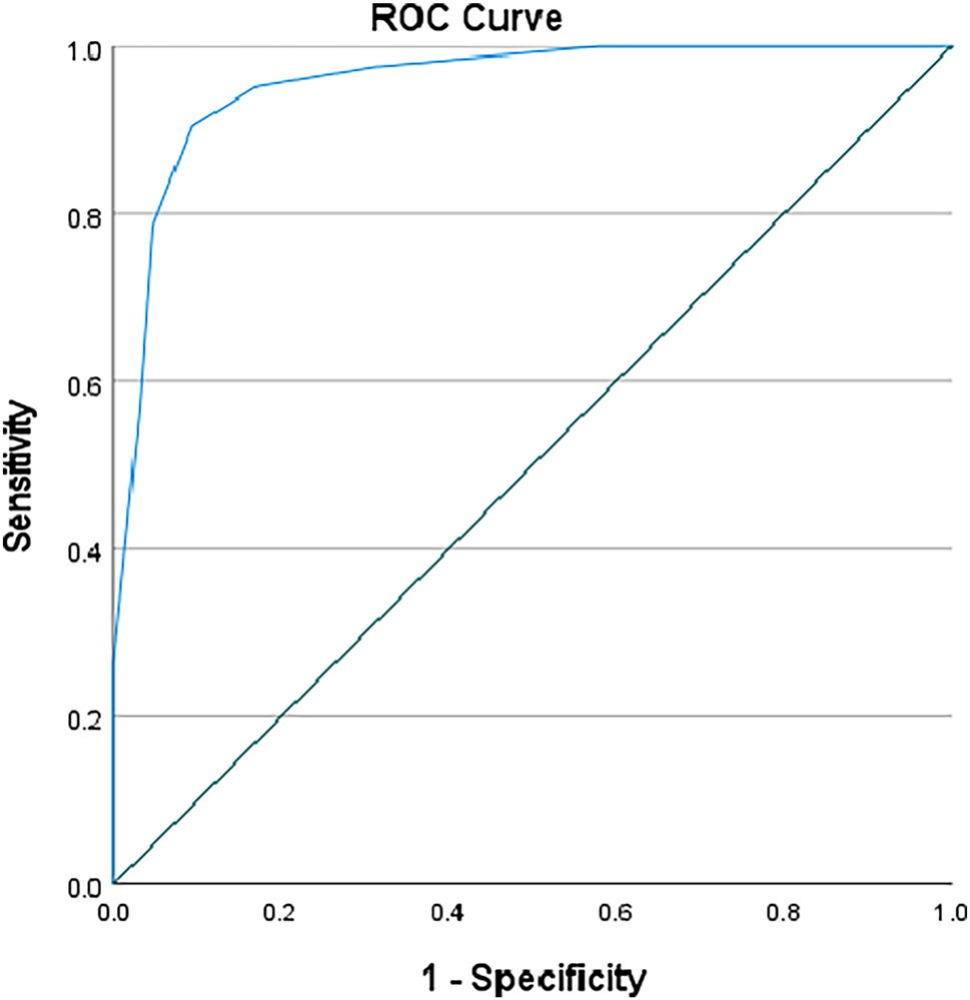
ROC curve analysis comparing the HD-DT-C barometer results with the HADS-T cutoff score ≥ 15 for clinically relevant distress

*Diagnostic accuracy and concurrent validity* The AUC results showed that the HD-DT-C barometer had an excellent discrimination value (*AUC* = 0.956, 95% CI 0.919–0.992; *p* < 0.001) (Fig. 2).

Online Resource 8 exhibits the results of sensitivity and (1-)specificity for each HD-DT-C barometer cutoff point computed against the HADS-T. Cutoffs of 5 and 6 on the HD-DT-C barometer were inspected for their diagnostic accuracy, as they both maximized sensitivity and specificity (Table 4) [32, 41]. In this sense, a cutoff value of 6 (5.50) yielded good to excellent sensitivity at 90.5% (95% CI 0.779–0.962) and specificity at 90.6% (95% CI 0.810–0.956) with positive and negative predictive values of 86.4% and 93.5%, respectively. Similarly, a cutoff value of 5 (4.50) showed good to excellent sensitivity at 95.2% (95% CI 0.842–0.987) and specificity at 82.8% (95% CI 0.718–0.901) with positive and negative predictive values of 78.4% and 96.4%, respectively.

**Table 4:** ROC analysis results for the HD-DT-C barometer against HADS-T (reference standard)

	AUC^a^ [95% CI]	HD-DT-F cutoff	Sensitivity [95% CI]	Specificity [95% CI]	PPV%	NPV%	Youden Index^b^	Utility Index^c^
HADS-total distress	0.956 [0.919–0.992]	6	0.905 [0.779–0.962]	0.906 [0.810–0.956]	86.4	93.5	0.81	0.781 [Good]
		5	0.952 [0.842–0.987]	0.828 [0.718–0.901]	78.4	96.4	0.78	0.747 [Good]

*AUC* Area Under the Curve, *CI* Confidence Interval, *HADS* Hospital Anxiety and Depression Scale, *PPV* Positive Predictive Value, *NPV* Negative Predictive Value

^a^AUC values were interpreted as follows: 0.5–0.7 = low, 0.7–0.9 = moderate, and 0.9–1.0 = high accuracy [41]

^b^Youden Index (*J*) results can vary between 0 and 1: values close to 0 indicate that a diagnostic test gives the same proportion of positive results for groups with and without the condition, while values closer to 1 suggest that there are no false positives or false negatives [34]

^c^Clinical Utility Index (*CUI* +) results were interpreted as follows: excellent utility ≥ 0.81, good utility ≥ 0.64, satisfactory utility ≥ 0.49, and poor utility < 0.49 [33]

The *J* index confirmed that both cutoffs can accurately identify caregivers with clinically relevant distress (*J*_cutoff ≥6_ = 0.81; *J*_cutoff ≥5_ = 0.78). The values obtained for the *CUI* were also suggestive of good utility for both cutoff scores (*CUI* + _cutoff ≥6_ = 0.781; *CUI* + _cutoff ≥5_ = 0.747).

*Convergent construct validity* The convergent validity tests of the European Portuguese version of the HD-DT-C (Table 5) revealed positive correlations of large magnitude [42] with the HADS-A (*r* = 0.860 for the barometer and *r* = 0.729 for the checklist; *p* < 0.001), HADS-D (*r* = 0.798 for the barometer and *r* = 0.673 for the checklist; *p* < 0.001), and ZBI total score (*r* = 0.556 for the barometer and *r* = 0.669 for the checklist; *p* < 0.001). Large magnitude coefficients with a negative direction were found for the correlations with related constructs from the WHOQOL-BREF, namely the ‘psychological health domain’ (*r* = − 0.644 for the barometer and *r* = − 0.603 for the checklist; *p* < 0.001). These results were in accordance with the predetermined hypotheses for the construct validity of the HD-DT-C (cf. Table 2).

**Table 5:** Validity tests of the HD-DT-C using Pearson’s product-moment correlation coefficient (*r*)

	Distress barometer	Checklist of difficulties and/or concerns^a^
Distress barometer	–	0.744
HADS-Total distress	0.874	0.739
HADS-Anxiety subscale	0.860	0.729
HADS-Depression subscale	0.798	0.673
WHOQ-BREF Psychological health domain	− 0.644	− 0.603
ZBI Total Score	0.556	0.669

Pearson’s *r* values were interpreted as small = 0.10, medium = 0.30, and large = 0.50 magnitude correlations [42]. All *p* < 0.01

*HADS* Hospital Anxiety and Depression, *WHOQ-BREF*  World Health Organization Quality of Life Instruments – BREF, *ZBI* Zarit Burden Interview

^a^Validity tests of the HD-DT-C checklist (categorical variables) were performed for the total number of difficulties and/or concerns (numerical variable) pointed out by respondents

## Discussion

This study reported the development process, content validity, clinical utility, test–retest reliability, and preliminary concurrent and convergent validity of the HD-DT-C, a new tool for screening psychological distress among family caregivers of adults on hemodialysis. Overall, the findings showed that the HD-DT-C is a valid, reliable, practical, and acceptable measure to quickly assess the presence of distress and its sources in this population.

The potential contribution of the HD-DT-C to research and clinical practice is particularly relevant given the scarcity of health-related outcome measures specifically designed for hemodialysis caregivers [16]. Compared to other general distress screening tools such as the HADS [26] (used as a reference standard in the present investigation), the HD-DT-C allows for a more comprehensive assessment of the idiosyncratic nature of hemodialysis-related caregiving stressors, which may increase its clinical utility in nephrology centers [17, 18]. Additional advantages of choosing the HD-DT-C over other screening instruments are related to its brevity, comprehensibility, and ease of administration and/or scoring, characteristics that may facilitate its dissemination and routine use in traditionally overburdened and resource-poor healthcare contexts, such as dialysis units [15]. Still, the use of this measure alone to detect the presence of clinically relevant psychological distress should be undertaken with caution due to the increased risk of identifying false-positive cases, a caveat generally associated with ultra-brief tools such as the HD-DT-C [44–46].

In both clinical practice and research, the costs and burden of identifying false positives using this new measure can be mitigated if distress screening is followed by a review of the scores and identification of the most predominant difficulties and/or concerns from the checklist [44, 45]. The large magnitude correlations (*r* > 0.50; indicative of strong convergent validity) with established screening measures of self-reported psychological health (WHOQOL-BREF), caregiver burden (ZBI), and anxiety (HADS-A) and depression (HADS-D) symptoms, indicate that the HD-DT-C can be used as a starting point for a more detailed psychological assessment [46]. In addition, the open question that was added to the tool, inquiring caregivers about their desire to receive support in dealing with the different sources of distress, may help ensure that only those who need/want to receive professional help are referred to the most appropriate support services (e.g., psychologist, psychiatrist, social worker) [44, 45].

In line with this, it should be noted that the choice of the most appropriate cutoff score for the HD-DT-C barometer (6 vs. 5) to detect clinically relevant psychological distress needs to consider the specific context in which the measure is intended to be applied (e.g., the tolerance for false positive screenings and resources available to meet the psychosocial needs of caregivers identified as 'distressed') [32, 47]. For instance, classifying as 'distressed' those who are actually 'non-cases' may contribute to an overestimation of the prevalence of psychological distress in hemodialysis caregivers and/or discredit interventions that may be more effective for people who are truly at risk for worse health outcomes (i.e., true positives) [48]. Consequently, the adoption of different cutoff points depending on distress screening goals has been previously recommended in studies reporting the validity of distress thermometers as a means of overcoming the shortcomings of high false positive rates (~ 20%) [49, 50]. In general, choosing a lower cutoff score (≥ 5) is more accepted in clinical practice to maximize the sensitivity of a measure to detect positive cases [51]; however, for research purposes, it may be advisable to opt for a higher cutoff score (≥ 6), as increased identification of false positives may limit the interpretation and transferability of results from observational and intervention studies [51, 52].

Though systemic distress screening (and management) continues to be a challenge for most renal care settings around the world [10, 11, 53], it is worth mentioning the potential benefits of improving the identification and assistance provided to ‘distressed’ caregivers [15, 54]. In this regard, some important insights can be obtained from studies carried out in oncology centers where this triage has been successfully implemented [54, 55]. Overall, research in this context has shown that supporting cancer caregivers not only helps reduce their levels of psychological distress but also enhances the cared-for person’s ability to effectively cope with treatment-related unpleasant symptoms such as pain, sleep problems, and reduced sexual functioning [56]. In turn, it has been proposed that caregivers who experience high levels of distress perceive a greater burden [57, 58], which may decrease their ability to perform caregiving activities and, consequently, worsen the health of the cared-for person and increase their need for medical attention [9].

While there is still limited knowledge on the interdependence between the physical and mental health trajectories of people living with kidney failure and their families, addressing the concerns and needs of hemodialysis caregivers may be crucial to improving quality of life outcomes for both members of the dyad [5, 59]. Due to the low presence of mental health professionals in nephrology centers [15, 43, 53], dyadic interventions may offer a promising approach to help balance the cost-effectiveness of providing psychosocial support to this population, as suggested by recent meta-analytical studies proving the effectiveness of family-based approaches in other clinical settings (e.g., cancer, respiratory diseases, rheumatic arthritis) [60]. More work is thus needed to understand the feasibility, acceptability, and positive impacts of organizing dyadic initiatives in dialysis care contexts [5, 59].

### Limitations

Some of the downsides of the present investigation are related to its cross-sectional design, which did not allow the testing of other measurement properties of the HD-DT-C (e.g., responsiveness, minimally important differences). This preliminary validation study was also performed with a relatively small convenience sample of 106 family caregivers recruited from dialysis units in Portugal, the country with the highest incidence of kidney failure in Europe and one of the highest prevalence rates of people on renal therapy in the world, according to the 2022 Annual Report of the United States Renal Data System [61]. Due to limited resources, the a priori estimated number of participants (*n* = 114) was not reached and, therefore, a larger sample size would be desirable for future research aimed at further attesting to the ability of HD-DT-C to discriminate between people with and without psychological distress. Another potential caveat to sampling bias is related to the representativeness of the perspectives and experiences of renal nurses, nephrologists, and family caregivers involved in developing and testing the psychometric properties of the HD-DT-C. For instance, most members of the target population who participated in the item generation were women and the new measure was pretested only with middle-aged people. Notwithstanding, these sociodemographic characteristics follow the global trend that family caregiving is mostly carried out by women and less frequently by younger adults [66]. The limited representation of men who provide informal care to a family member or friend with kidney failure may also be associated with epidemiological characteristics of this chronic disease since the incidence of people on hemodialysis is higher among men than women [67]. All things considered, caution is needed when generalizing the preliminary validation results found for the barometer cutoff values, the prevalence of distress and its sources in hemodialysis caregivers, and the acceptability of the tool for other culturally distinct samples. It is also worth declaring that a new instrument measuring the burden of caregivers in this context was recently developed—the Caregiver Burden Questionnaire for Family Caregivers of Hemodialysis Patients [16]. This tool was not identified in the literature search that informed the generation of the HD-DT-C checklist since, at that time (until June 2022), it had not yet been published.

## Conclusion

The HD-DT-C is a reliable, valid, practical, and acceptable measure to quickly identify self-reported psychological distress among people caring for a family member or friend on hemodialysis. The use of brief measures that allow rapid assessment of distress and its sources, such as the HD-DT-C, can be seen as a crucial step in raising awareness of the need to prepare and/or reinforce psychosocial screening and/or interventions targeting family caregivers in nephrology centers around the world.

### Supplementary Information

Below is the link to the electronic supplementary material.

Online Resource 1: Multidimensional model of clinical utility. Supplementary file1 (DOCX 23 KB)

Online Resource 2: HD-DT-C Acceptability Questionnaire. Supplementary file2 (DOCX 27 KB)

Online Resource 3: The Hemodialysis Distress Thermometer – Caregiver Version (HD-DT-C). Supplementary file3 (DOCX 48 KB)

Online Resource 4. Identification of hemodialysis-related caregiving stressors to inform the development of the HD-DT-C checklist. Supplementary file4 (DOCX 49 KB)

Online Resource 5. List of items tested for content validity by two feedback panels. Supplementary file5 (DOCX 43 KB)

Online Resource 6. Adjustments made to the HD-DT-C after focus group interviews with feedback panels. Supplementary file6 (DOCX 27 KB)

Online Resource 7. Intra-rater values for the barometer and categorical items of the European-Portuguese version of the HD-DT-C during test-retest reliability. Supplementary file7 (DOCX 32 KB)

Online Resource 8. Sensitivity and (1-)specificity for each cutoff point on the HD-DT-C barometer against the HADS-T cutoff score ≥15 for total distress. Supplementary file8 (DOCX 25 KB)

## Data Availability

The data that support the findings of this study are available from the corresponding author,H.S., upon reasonable request.
