# Supplementary material for: The Hemodialysis Distress Thermometer for Caregivers (HD-DT-C): development and testing of the psychometric properties of a new tool for screening psychological distress among family caregivers of adults on hemodialysis
Source: Qual Life Res. 2024 Mar 7;33(6):1513–26. doi: 10.1007/s11136-024-03627-x (PMC11116227; doi:10.1007/s11136-024-03627-x)
Supplement: Supplementary file 1 — Online Resource 1: Multidimensional model of clinical utility. Supplementary file1 (DOCX 23 KB) [file 11136_2024_3627_MOESM1_ESM.docx]

**Online Resource 1.** Smart's (2006) multidimensional model of clinical utility (Smart, 2006^1^; Sousa et al., 2021b^2^).

| **Evaluating the clinical utility of the HD-DT-C in renal care setting using Smart’s multidimensional model** | **Components** | **Aspects to consider** | **Questions to ask** |
| --- | --- | --- | --- |
|  | **Accessibility** | **Resources and economic**  **implications** | *How much it costs?* |
|  |  | **Availability** | *Where and how to access it?*  *Is there technical support to clarify doubts and prevent misuse?* |
|  | **Practicality** | **Functional** | *Is it complete or a work in progress?* |
|  |  | **Suitable** | *Does it respond to the needs of the context?* |
|  |  | **Training or knowledge** | *Do health professionals have the necessary training and abilities to apply/use it?* |
|  | **Appropriateness** | **Effective** | *Is there formal evidence of its appropriateness in similar contexts?*  *Will it cause disruptions to current work or care?* |
|  |  | **Relevant** | *How will it imply the existent treatment?*  *Is it important for clinical decision-making?* |
|  | **Acceptability** | **To the dialysis team** | *Are there any ethical, legal, social, or psychological concerns that may interfere with practice or treatment? (e.g., time, perception of its usefulness)* |
|  |  | **To people on dialysis** | *Are there any ethical, legal, social, or psychological concerns that may interfere with practice or treatment? (e.g., time, perception of its usefulness) Is it easy to complete?* |

^1^ Smart A. (2006). A multi-dimensional model of clinical utility. *International Journal for Quality in Health Care, 18*, 377–382. doi:10.1093/intqhc/mzl034

^2^ Sousa, H., Oliveira, J., Figueiredo, D. and Ribeiro, O. (2021). The clinical utility of the Distress Thermometer in non-oncological contexts: A scoping review. *Journal of Clinical Nursing, 30*, 2131–2150. doi:10.1111/jocn.15698
