# Supplementary material for: The Hemodialysis Distress Thermometer for Caregivers (HD-DT-C): development and testing of the psychometric properties of a new tool for screening psychological distress among family caregivers of adults on hemodialysis
Source: Qual Life Res. 2024 Mar 7;33(6):1513–26. doi: 10.1007/s11136-024-03627-x (PMC11116227; doi:10.1007/s11136-024-03627-x)
Supplement: Supplementary file 2 — Online Resource 2: HD-DT-C Acceptability Questionnaire. Supplementary file2 (DOCX 27 KB) [file 11136_2024_3627_MOESM2_ESM.docx]

**Online Resource 2.** HD-DT-C Acceptability Questionnaire.

| **The Hemodialysis Distress Thermometer for Caregivers (HD-DT-C)**  **Acceptability Questionnaire*** | | | | | | | | | |
| --- | --- | --- | --- | --- | --- | --- | --- | --- | --- |
| **The Hemodialysis Distress Thermometer for Caregivers (HD-DT-C) is a newly developed measure that aims to assess the level of psychological distress and the main difficulties and/or concerns experienced by caregivers of adults on hemodialysis.**  **Please mark with an X the option that best fits your opinion about the HD-DT-C:** | | | | | | | | | |
| 1. **How much effort did it take you to complete the measure?** | | | | | | | | | |
| **No effort** | | **Little effort** | | | **Some effort** | | | **Intense effort** | |
| 1. **To what extent did you feel comfortable answering the measure?** | | | | | | | | | |
| **Very comfortable** | **Comfortable** | | | **Moderately comfortable** | | **Uncomfortable** | | | **Very uncomfortable** |
| 1. **To what extent did you consider the response time of the measure to be acceptable?** | | | | | | | | | |
| **Very acceptable** | **Acceptable** | | | **Moderately acceptable** | | **Not acceptable** | | | **Very unacceptable**  **(it took me too long)** |
| 1. **To what extent did you find it easy to understand the measure items (checklist of difficulties and/or concerns)?** | | | | | | | | | |
| **Very easy** | **Easy** | | | **Moderately easy** | | **Hard** | | | **Very hard** |
| 1. **How easy did you find it to quantify your level of psychological distress from 0 to 10?** | | | | | | | | | |
| **Very easy** | **Easy** | | | **Moderately easy** | | **Hard** | | | **Very hard** |
| 1. **To what extent do you consider this measure adequate for tracking the difficulties and/or concerns of caregivers of adults on hemodialysis?** | | | | | | | | | |
| **Very adequate** | **Adequate** | | | **Moderately adequate** | | **Inadequate** | | | **Very inadequate** |
| 1. **To what extent do you consider this measure useful for tracking the difficulties and/or concerns of caregivers of adults on hemodialysis?** | | | | | | | | | |
| **Very useful** | **Useful** | | | **Moderately useful** | | **Useless** | | | **Very useless** |
| 1. **To what extent do you consider this measure to be practical for tracking the difficulties and/or concerns of caregivers of adults on hemodialysis?** | | | | | | | | | |
| **Very practical** | **Practical** | | | **Moderately practical** | | **Unpractical** | | | **Very unpractical** |
| 1. **If suggested by the dialysis unit where your family member receives hemodialysis treatment, would you agree to complete this measure regularly? (e.g., once a year)** | | | | | | | | | |
| **Yes** | | | **Uncertain** | | | | **No** | | |
| **Please, give us your opinion about the measure and any indication that you consider important about its structure, content, and/or readability of the items (for example, difficulties and/or concerns that would be important to add, wording alternatives for items that you considered difficult to understand)** | | | | | | | | | |

***** This questionnaire was developed in Portuguese and translated into English for the purposes of the present study.
