# Supplementary material for: The Hemodialysis Distress Thermometer for Caregivers (HD-DT-C): development and testing of the psychometric properties of a new tool for screening psychological distress among family caregivers of adults on hemodialysis
Source: Qual Life Res. 2024 Mar 7;33(6):1513–26. doi: 10.1007/s11136-024-03627-x (PMC11116227; doi:10.1007/s11136-024-03627-x)
Supplement: Supplementary file 3 — Online Resource 3: The Hemodialysis Distress Thermometer – Caregiver Version (HD-DT-C). Supplementary file3 (DOCX 48 KB) [file 11136_2024_3627_MOESM3_ESM.docx]

**HEMODIALYSIS DISTRESS THERMOMETER**

**- Caregiver Version**

**HD-DT-C**

**EMOTIONAL DISTRESS IS AN UNPLEASANT EXPERIENCE OF A PHYSICAL, PSYCHOLOGICAL, SPIRITUAL, OR SOCIAL NATURE, WHICH CAN INFLUENCE THE WAY YOU THINK, FEEL, OR ACT TOWARD THE HEMODIALYSIS TREATMENT OF YOUR FAMILY MEMBER/LOVED ONE WITH KIDNEY FAILURE.**

**Circle the number from 0 to 10 that best describes the level of emotional distress (example: hopelessness, sadness, anxiety, worry) you have been feeling throughout this week, including today:**

| **10 - Extreme emotional distress** | 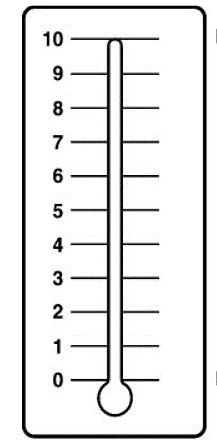 |
| --- | --- |
| **0 – No emotional distress** |  |

**Date: ___/___/___**

**Name: ________________________________________________**

**Mark with an X the difficulties and/or concerns you have experienced concerning the care/support you provide to your family member/loved one with kidney failure, throughout this week, including today:**

**PHYSICAL DIFFICULTIES AND/OR CONCERNS:**

🞏 Tiredness and/or fatigue

🞏 Sleep changes

🞏 Changes in sexual life/intimacy

🞏 Changes in physical ability (e.g., difficulty doing household chores, moving around)

**PSYCHOLOGICAL DIFFICULTIES AND/OR CONCERNS:**

🞏 Changes in memory and/or concentration

🞏 Sadness and/or depression

🞏 Nervousness and/or anxiety

🞏 Feelings of grief and/or loss

🞏 Feelings of guilt (e.g., feeling I should do more for my family member/loved one on dialysis, feeling I should pay more attention to other family members)

🞏 Feeling overwhelmed by the responsibilities of caring for my family member/loved one

🞏 Concern about the health of my family member/loved one

🞏 Fear that I will no longer be able to care for my family member/loved one

🞏 Concern about my family member/loved one's kidney transplant

🞏 Difficulty in dealing with my family member/loved one's negative feelings (e.g., anger, sadness, hopelessness)

🞏 Not knowing how to support (e.g., encourage, reassure) my family member/loved one

🞏 Difficulty in accomplishing my goals and life projects

**SOCIAL/FAMILY DIFFICULTIES AND/OR CONCERNS:**

🞏 Caring for my family member/loved one affects my social and/or family life (e.g., less time/availability for vacation, leisure, work)

🞏 Lack of family support in the distribution of caregiving responsibilities

**DIFFICULTIES AND/OR CONCERNS IN MANAGING CAREGIVING TASKS:**

🞏 Problems with my family member/loved one's transportation (e.g., to dialysis and/or medical appointments)

🞏 Dealing with my family member/loved one's resistance to treatment (e.g., to dialysis sessions, fluid and/or dietary restrictions)

🞏 Difficulty denying liquids when my family member/loved one feels thirsty

🞏 Difficulty knowing how much liquids my family member/loved one can drink daily

🞏 Being creative with meals so that my family member/loved one does not lose his/her appetite

🞏 Difficulty denying certain foods to my family member/loved one

🞏 Knowing what is (or is not) recommended for my family member/loved one's diet

🞏 Managing family meals and the care my family member/loved one needs with food

🞏 Taking care of my family member/loved one's vascular access (fistula or catheter)

🞏 Managing the different medications my family member/loved one takes

🞏 Lack of information about my family member/loved one's health status, treatments, and possible complications

🞏 Financial difficulties

**OTHER DIFFICULTIES AND/OR CONCERNS:**

**____________________________________________________________________________________________________________________________________________________________________________________________________________________________________________________________________________________________________________________________________________________________________________________________________________________________________________________________________________________________________________________________________________________________________________________________________________________________________________________________**

**WOULD YOU LIKE TO GET SUPPORT IN DEALING WITH ANY OF THE ABOVE DIFFICULTIES AND/OR CONCERNS? IF SO, PLEASE SPECIFY:**

**____________________________________________________________________________________________________________________________________________________________________________________________________________________________________________________________________________________________________________________________________________________________________________________________________________________________________________________________________________________________________________________________________________________________________________________________________________________________________________________________**
