# Supplementary material for: The Hemodialysis Distress Thermometer for Caregivers (HD-DT-C): development and testing of the psychometric properties of a new tool for screening psychological distress among family caregivers of adults on hemodialysis
Source: Qual Life Res. 2024 Mar 7;33(6):1513–26. doi: 10.1007/s11136-024-03627-x (PMC11116227; doi:10.1007/s11136-024-03627-x)
Supplement: Supplementary file 4 — Online Resource 4. Identification of hemodialysis-related caregiving stressors to inform the development of the HD-DT-C checklist. Supplementary file4 (DOCX 49 KB) [file 11136_2024_3627_MOESM4_ESM.docx]

**Online Resource 4.** Identification of hemodialysis-related caregiving stressors to inform the development of the HD-DT-C checklist.

| **Stressors** | **Identified in:** | | | |
| --- | --- | --- | --- | --- |
|  | **Checklists of other distress thermometers** | **Items retrieved from the literature search** | **Transcribed interviews** | |
|  |  |  | **Family caregivers (*n*=32)** | **Dialysis care professionals (*n*=23)** |
| 1. Feelings of worthlessness or being a burden | X |  |  |  |
| 1. Feelings of grief and/or loss | X |  |  |  |
| 1. Anger | X | X |  |  |
| 1. Problems with changes in your appearance | X |  |  |  |
| 1. Loss or change of physical abilities | X |  |  |  |
| 1. Patient care has prevented me from exercising or having beneficial physical mobility |  | X |  |  |
| 1. Changes in appetite | X |  |  |  |
| 1. Pain | X |  |  |  |
| 1. Taking care of the patient has caused me physical pain |  | X |  |  |
| 1. Concerned with your ability to have children | X |  |  |  |
| 1. Problems with sleep | X |  |  |  |
| 1. I am not satisfied with the quality of my sleep |  | X |  |  |
| 1. I don’t sleep well at night |  | X |  |  |
| 1. Problems with memory or concentration | X |  |  |  |
| 1. I feel less focused |  | X |  |  |
| 1. Problems with sexual health | X |  |  |  |
| 1. Difficulty denying certain foods to your family member |  |  | X |  |
| 1. Needing more information about what is recommended for the diet of a person on hemodialysis |  |  | X | X |
| 1. Being creative with meals so that the cared-for person does not lose appetite |  |  | X |  |
| 1. Not knowing the recommended amount of fluids for an adult on hemodialysis to ingest daily |  |  | X | X |
| 1. Difficulty denying fluids when your family member is thirsty |  |  | X | X |
| 1. Difficulty managing the administration (and dosages) of different medications |  |  | X | X |
| 1. Problems with access to medicine | X |  |  |  |
| 1. Doubts about how to help the family member on dialysis with the vascular access hygiene, hydration, and care |  |  | X |  |
| 1. Doubts on how to manage fistula-related complications |  |  | X | X |
| 1. Problems with transportation | X |  |  |  |
| 1. Problems with treatment decisions | X |  |  |  |
| 1. My efficiency at work or home has been reduced |  | X |  |  |
| 1. Problems with housing conditions | X |  |  |  |
| 1. Having all the responsibility for caring |  | X |  |  |
| 1. Lack of support from close family members with whom they wished to share caregiving responsibilities |  |  | X |  |
| 1. Other family members do not accompany me in caring |  | X |  |  |
| 1. Strained relationship with secondary caregivers |  |  | X |  |
| 1. Other family members and friends don’t help me move my patient and bring him to the hospital |  | X |  |  |
| 1. I do not feel the sympathy and support of my loved ones |  | X |  |  |
| 1. I want to assign taking care of my patient to another person. |  | X |  |  |
| 1. I wish to leave the care of your family member to someone else |  | X |  |  |
| 1. My family member does not seem to appreciate all I do for him/her |  | X |  |  |
| 1. My family member doesn’t consider my feelings |  | X |  |  |
| 1. I don’t feel valued by others because of taking care of the patient |  | X |  |  |
| 1. It seems that others have inappropriate care expectations from me |  | X |  |  |
| 1. I am worried about others’ judgment about how my patient care works |  | X |  |  |
| 1. Feeling overwhelmed by caregiving responsibilities |  | X |  | X |
| 1. Fear of not being able to continue caring for your family member |  | X |  |  |
| 1. Problems with childcare | X |  |  |  |
| 1. Taking care of my patient has led to improper handling of other family members |  | X |  |  |
| 1. I feel guilty that taking care of my patient prevents me from paying attention to the affairs of other members of my family |  | X | X |  |
| 1. Taking care of my patient has left me with no opportunity to take care of other matters of life, including education and work |  | X |  |  |
| 1. Problems taking care of others | X |  |  |  |
| 1. I feel guilty when my patient’s condition worsens |  | X |  |  |
| 1. I criticize myself |  | X |  |  |
| 1. I blame myself for what happened |  | X |  |  |
| 1. Feeling like you should do more for your family member |  | X |  |  |
| 1. Feeling like you could do a better job caring for your family member |  | X |  |  |
| 1. Sometimes I say things to family member that wish I had never said |  | X |  |  |
| 1. I am afraid that the way I take care of my patient harms him/her |  | X |  |  |
| 1. As a result of the dialysis patient care, I feel the house is psychologically disturbing |  | X |  |  |
| 1. Problems taking care of yourself | X |  |  |  |
| 1. Not having enough time for yourself |  | X |  |  |
| 1. Taking care of my patient has overshadowed my life |  | X |  |  |
| 1. Taking care of my patient has made me give up my fun and work |  | X |  |  |
| 1. Due to the multiplicity of tasks, I have a conflict in deciding between the role of care and doing other important things in life |  | X |  |  |
| 1. Feeling like you’ve lost control over your life |  | X |  |  |
| 1. I am not satisfied with my social relationships |  | X |  |  |
| 1. Dealing with the negative impacts of caregiving on personal, family, and social goals |  |  |  | X |
| 1. Excessive hospital visits have disrupted other aspects of my life |  | X |  |  |
| 1. Patient care has made me forget my interests and preferences |  | X |  |  |
| 1. I don’t hold a favorable view towards life because of taking care of a dialysis patient |  |  |  |  |
| 1. I can’t do all the things I used to do |  | X |  |  |
| 1. Uncertainty about what to do for your family member |  | X |  |  |
| 1. Not knowing how to deal with the negative feelings (e.g., distress, anger, thoughts on withdrawing from treatment) of the person on dialysis |  |  | X | X |
| 1. Difficulty dealing with your family member resistance to fluid and dietary restrictions |  |  | X | X |
| 1. Difficulty dealing with your family member resistance to adherence to hemodialysis sessions |  |  | X | X |
| 1. Worry or anxiety | X | X |  |  |
| 1. Having a sense of strain |  | X |  |  |
| 1. My mind is always engaged with my patient’s illness |  | X |  |  |
| 1. I’m worried about trying to leave my patient alone for a moment |  | X |  |  |
| 1. I feel being ignored |  | X |  |  |
| 1. My level of distress has increased |  | X |  |  |
| 1. Depression or sadness | X | X |  |  |
| 1. Constantly watching my patient’s condition makes me sad |  | X |  |  |
| 1. I do not try to look at my problems from a positive perspective |  | X |  |  |
| 1. I do not think about the good things that have happened in my life |  | X |  |  |
| 1. Loss of interest or enjoyment | X |  |  |  |
| 1. I suffer from my patients’ suffering imposed by illness and dialysis |  | X |  |  |
| 1. I experience overwhelming stress in caring for my patient |  | X |  |  |
| 1. I feel that my patient is too dependent on me |  | X |  |  |
| 1. Caregiving has negatively impacted privacy |  | X |  |  |
| 1. Problems in your relationship with your spouse or partner | X |  |  |  |
| 1. Problems in your relationship with your children | X |  |  |  |
| 1. Problems in your relationship with your family members | X |  |  |  |
| 1. I mentally feel tired because of dialysis patient care |  | X |  |  |
| 1. Fatigue | X |  |  |  |
| 1. My health has worsened |  | X |  |  |
| 1. Taking care of the patient has made me downgrade my health |  | X |  |  |
| 1. Lower motivation when fatigued |  | X |  |  |
| 1. Fatigue interferes with physical functioning |  | X |  |  |
| 1. Fatigue causes problems |  | X |  |  |
| 1. Fatigue interferes with carrying out certain duties and responsibilities (such as caregiving tasks) |  | X |  |  |
| 1. Fatigue interferes with work, family, or social life |  | X |  |  |
| 1. Dealing with the patient’s frustration with his illness leads to mental fatigue in me |  | X |  |  |
| 1. Taking care of my patient and multiple tasks has caused me a lot of stress and fatigue |  | X |  |  |
| 1. The lack of welfare facilities in the hospital makes me tired |  | X |  |  |
| 1. I feel tired and frustrated as I have to go to the hospital so many times |  | X |  |  |
| 1. I often feel physically exhausted |  | X |  |  |
| 1. Feeling frustrated and/or hopeless about the disease/treatments of the cared-for person |  | X |  |  |
| 1. Fear of not having enough money to care for your family member |  | X |  |  |
| 1. Problems with finances | X |  |  |  |
| 1. I have financial concerns about medicine, dialysis, and living expenses |  | X |  |  |
| 1. Not having enough food | X |  |  |  |
| 1. I am worried about my financial status |  | X |  |  |
| 1. Finances related to treatment are a source of stress |  | X |  |  |
| 1. Problems with insurance | X |  |  |  |
| 1. The costs of the patient’s regular travel to the hospital are not covered by insurance |  | X |  |  |
| 1. Fearing the future regarding your family member’s health condition |  | X |  | X |
| 1. Difficulties in dealing with the progressive decline of the physical and cognitive function of the person being cared for |  |  |  | X |
| 1. I am afraid of the indefinite future I have for the patient care |  | X |  |  |
| 1. I am afraid that my patient’s condition is getting worse |  | X |  |  |
| 1. I have many questions about my patient’s future condition |  | X |  |  |
| 1. I am worried about the future status of my patient |  |  |  |  |
| 1. I am confused because I do not know what will happen to my patient |  | X |  |  |
| 1. Loneliness | X |  |  | X |
| 1. I feel lonely and helpless most of the time |  | X |  |  |
| 1. Problems with your relationship with friends or coworkers | X |  |  |  |
| 1. At work, they don’t work with me to take care of my patient and bring him to the hospital |  | X |  |  |
| 1. Problems with your family member’s healthcare team | X |  |  |  |
| 1. When I go to the doctor’s office, he does not guide me in a good mood and does not allocate appropriate time in how to take care of my patient |  | X |  |  |
| 1. At the hospital, the nurses do not pay enough attention to my and my patient’s requests |  | X |  |  |
| 1. Problems with tobacco use | X |  |  |  |
| 1. Problems with substance use | X | X |  |  |
| 1. Concerned about your sense of meaning or purpose | X |  |  |  |
| 1. I don’t plan or set goals to do anything |  | X |  |  |
| 1. Dialysis patient care limits my decision about my future |  | X |  |  |
| 1. Concerned about changes in my faith or beliefs | X |  |  |  |
| 1. Feeling conflicted between spiritual/religious beliefs and your family members’ treatments | X |  |  |  |
| 1. Concerned about your relationship with the sacred | X |  |  |  |
| 1. Concerned about your spiritual/religious rituals | X |  |  |  |
| 1. There is no formal training for me in the hospital despite my frequent hospitalizations |  | X |  |  |
| 1. There is no radio or television education on how to care for my patient |  | X |  |  |
| 1. Books that simply describe the disease, its symptoms, and how to control it are not available |  | X |  |  |
| 1. There are no specialized centers for hemodialysis patients to help and educate me and my patient |  | X |  |  |
| 1. Lack of written training by the medical system confuses me in dealing with my patient’s condition |  | X |  |  |
| 1. Due to the lack of information about my patient’s condition, I frequently see different doctors |  | X |  |  |
| 1. Transportation to treatment and doctors’ appointments is a source of stress |  | X |  |  |
| 1. Time management is a source of frustration |  | X |  |  |
| 1. My daily life has lots of fluctuations due to the unstable conditions of my patient |  | X |  |  |
| 1. As my family member became sick, my need for medical and pharmaceutical treatment was further enhanced |  | X |  |  |
| 1. I am worried about the complications of dialysis and the improper functioning of the dialysis machines |  | X |  |  |
| 1. I am embarrassed for my patients’ behaviors, which are due to kidney failure |  | X |  |  |
| 1. Concerns about the kidney transplant of the person under treatment |  |  |  | X |
