# Supplementary material for: The Hemodialysis Distress Thermometer for Caregivers (HD-DT-C): development and testing of the psychometric properties of a new tool for screening psychological distress among family caregivers of adults on hemodialysis
Source: Qual Life Res. 2024 Mar 7;33(6):1513–26. doi: 10.1007/s11136-024-03627-x (PMC11116227; doi:10.1007/s11136-024-03627-x)
Supplement: Supplementary file 5 — Online Resource 5. List of items tested for content validity by two feedback panels. Supplementary file5 (DOCX 43 KB) [file 11136_2024_3627_MOESM5_ESM.docx]

**Online Resource 5.** List of items tested for content validity by two feedback panels: expert panel (*n*=9 dialysis care professionals) and target population group (*n*=10 hemodialysis caregivers). Clusters and their definitions are also displayed. Each cluster represents a section of the HD-DT-C checklist.

| Clusters (*n*=4) | Item* | I-CVI  Total | I-CVI Expert Panel | I-CVI  Target Population |
| --- | --- | --- | --- | --- |
| Physiological stressors  (*n*=6 items)  *This cluster encompasses negative physiological or physical responses and/or reactions to caregiving* | **Tiredness and/or fatigue** | 0.842 | 1.000 | 0.700 |
|  | **Pain** | 0.526 | 0.778 | 0.300 |
|  | **Sleep changes** | 0.895 | 1.000 | 0.800 |
|  | **Appetite changes** | 0.158 | 0.000 | 0.300 |
|  | **Changes in sexual life/intimacy** | 0.526 | 0.778 | 0.300 |
|  | **Changes in physical ability (e.g., difficulty doing household chores, mobility, and moving around)** | 0.474 | 0.778 | 0.200 |
| Psychological stressors  (*n*=18 items)  *This cluster includes negative emotional, cognitive, and perceptual (e.g., beliefs, roles, attitudes) responses and/or reactions to caregiving* | **Problems with tobacco and/or substance use** | 0.000 | 0.000 | 0.000 |
|  | **Changes in memory and/or concentration** | 0.789 | 0.778 | 0.800 |
|  | **Dealing with changes in my appearance** | 0.211 | 0.333 | 0.100 |
|  | **Feelings of grief and/or loss** | 0.526 | 0.778 | 0.300 |
|  | **Feeling of uselessness** | 0.421 | 0.778 | 0.100 |
|  | **Feelings of guilt (e.g., feeling I should do more for my family members, on dialysis, feeling I should pay more attention to other family members)** | 0.684 | 1.000 | 0.400 |
|  | **Sadness and/or depression** | 1.000 | 1.000 | 1.000 |
|  | **Nervousness and/or anxiety** | 0.947 | 1.000 | 0.900 |
|  | **Loneliness** | 0.474 | 0.556 | 0.400 |
|  | **Anger and/or frustration** | 0.263 | 0.333 | 0.200 |
|  | **Concern about my family member’s health** | 1.000 | 1.000 | 1.000 |
|  | **Feeling overwhelmed by the responsibilities of caring for my family member** | 0.947 | 1.000 | 0.900 |
|  | **Fear that I will no longer be able to care for my family member** | 0.789 | 0.778 | 0.800 |
|  | **Concern about my ability to have children** | 0.000 | 0.000 | 0.000 |
|  | **Difficulty in dealing with my family member/loved one's negative feelings (e.g., anger, sadness, hopelessness)** | 1.000 | 1.000 | 1.000 |
|  | **Not knowing how to support (e.g., encourage, reassure) my family member** | 0.895 | 1.000 | 0.800 |
|  | **Difficulty in accomplishing my goals and life projects** | 0.579 | 0.333 | 0.800 |
|  | **Difficulty dealing with changes in my beliefs or faith** | 0.000 | 0.000 | 0.000 |
| Social stressors  (*n*=6 items)  *This cluster gathers social and relational experienced caregivers* | **Caring for my family member affects my social and/or family life (e.g., less time/availability for vacation, leisure, work)** | 0.947 | 1.000 | 0.900 |
|  | **Caring for my family member affects my relationship with other family members (e.g., children, adult children, siblings, spouse/partner, parents)** | 0.421 | 0.778 | 0.100 |
|  | **Caring for my family member affects my relationships with friends or co-workers** | 0.684 | 0.778 | 0.500 |
|  | **Difficulty in the relationship with my family member’s health team** | 0.421 | 0.778 | 0.100 |
|  | **Difficulty fulfilling family responsibilities (e.g., taking care of children, grandchildren)** | 0.526 | 0.778 | 0.300 |
|  | **Lack of family support in the distribution of caregiving responsibilities** | 0.842 | 0.667 | 1.000 |
| Stressors related to caregiving tasks^1^ (*n*=18 items)  *This cluster represents all concerns and difficulties associated with hemodialysis-specific caregiving activities* | **Problems with my housing conditions** | 0.474 | 0.778 | 0.200 |
|  | **Problems accessing my family member's medications** | 0.316 | 0.556 | 0.100 |
|  | **Problems with my family member/loved one's transportation (e.g., to dialysis and/or medical appointments)** | 0.737 | 0.778 | 0.700 |
|  | **Financial difficulties** | 0.789 | 1.000 | 0.600 |
|  | **Difficulty managing responsibilities/tasks at my job** | 0.684 | 0.778 | 0.600 |
|  | **Difficulty making decisions regarding my family member's treatment** | 0.474 | 0.778 | 0.200 |
|  | **Dealing with my family member’s resistance to treatment (e.g., to dialysis sessions, fluid and/or dietary restrictions)** | 1.000 | 1.000 | 1.000 |
|  | **Difficulty denying certain foods to my family member** | 1.000 | 1.000 | 1.000 |
|  | **Knowing what is (or is not) recommended for my family member’s diet** | 0.895 | 1.000 | 0.800 |
|  | **Being creative with meals so that my family member does not lose his/her appetite** | 0.947 | 1.000 | 0.900 |
|  | **Managing family meals and the care my family member needs with food** | 0.947 | 1.000 | 0.900 |
|  | **Difficulty knowing how much liquids my family member can drink daily** | 0.947 | 1.000 | 0.900 |
|  | **Difficulty denying liquids when my family member feels thirsty** | 0.947 | 1.000 | 0.900 |
|  | **Difficulty managing the administration (and dosage) of different medications** | 0.789 | 1.000 | 0.600 |
|  | **Difficulty in caring for my family member’s vascular access** | 0.474 | 0.778 | 0.200 |
|  | **Difficulty in providing hygiene care to my family member (e.g., helping him/her take a shower, get dressed)** | 0.526 | 1.000 | 0.100 |
|  | **Not having enough time for myself** | 0.474 | 0.222 | 0.700 |
|  | **Lack of information about my family member's health status, treatments, and possible complications** | 1.000 | 1.000 | 1.000 |

* These items were translated for this table and do not correspond to the final American English translation and cultural adaptation of the measure.

^1^ Later referred to as ‘difficulties and/or concerns in managing caregiving tasks’ in the final American English version of the HD-DT-C.
