# Supplementary material for: The Hemodialysis Distress Thermometer for Caregivers (HD-DT-C): development and testing of the psychometric properties of a new tool for screening psychological distress among family caregivers of adults on hemodialysis
Source: Qual Life Res. 2024 Mar 7;33(6):1513–26. doi: 10.1007/s11136-024-03627-x (PMC11116227; doi:10.1007/s11136-024-03627-x)
Supplement: Supplementary file 6 — Online Resource 6. Adjustments made to the HD-DT-C after focus group interviews with feedback panels. Supplementary file6 (DOCX 27 KB) [file 11136_2024_3627_MOESM6_ESM.docx]

**Online Resource 6.** Adjustments made to the HD-DT-C* after focus group interviews with dialysis care professionals (expert panel; *n*=9) and hemodialysis caregivers (target population group; *n*=10).

| **Modifications in the items in need of revision due to wording** **adequacy concerns** | 'Difficulty managing the administration (and dosage) of different medications' was changed to ‘Managing the different medications my family member takes’ |
| --- | --- |
| **Synonyms of the Anglo-Saxon term 'distress' to incorporate in the barometer** | Circle the number from 0 to 10 that best describes the level of emotional distress (e.g., hopelessness, sadness, anxiety, worry) you have been feeling throughout this week, including today |
| **Definition of distress to be included at the beginning of the measure** | Emotional distress is an unpleasant experience of a physical, psychological, spiritual, or social nature, which can influence the way you think, feel, or act toward the hemodialysis treatment of your family member/loved one/cared-for person with kidney failure |
| **Synonymous of the Anglo-Saxon term ‘stressor’ to incorporate in the checklist** | Difficulties, concerns |
| **‘Marginal’ items considered not relevant (or better represented by another item already present in the HD-DT-C) that were removed from the checklist after focus group interviews (*n*=11)** | Pain; feeling of uselessness; caring for my family member affects my relationship with other family members (e.g., children, adult children, siblings, spouse/partner, parents); caring for my family member affects my relationships with friends or co-workers; difficulty in the relationship with my family member’s health team; difficulty fulfilling family responsibilities (e.g., taking care of children, grandchildren); problems with my housing conditions; difficulty managing responsibilities/tasks at my job; difficulty making decisions regarding my family member's treatment; difficulty in providing hygiene care to my family member (e.g., helping him/her take a shower, get dressed); not having enough time for myself |
| **‘Marginal’ items considered relevant and recovered during focus group interviews (*n*=7)** | Changes in sexual life/intimacy; changes in physical ability (e.g., difficulty doing household chores, mobility, and moving around); feelings of grief and/or loss; feelings of guilt (e.g., feeling i should do more for my family members on dialysis, feeling i should pay more attention to other family members); difficulty in accomplishing my goals and life projects; problems with my family member/loved one's transportation (e.g., to dialysis and/or medical appointments); difficulty in caring for my family member’s vascular access |
| **Establishment of the recall period for the barometer and checklist** | *‘Throughout this week, including today’* - this time interval was chosen considering the HADS (reference measure) time interval for assessing psychological distress |
| **Insertion of an open question that allows the identification of other stressors that were not present in the HD-DT-C checklist** | The item 'Other difficulties and/or concerns’ was added as an open question to allow a more comprehensive assessment of the difficulties experienced by caregivers |
| **Inclusion of an open question about the desire to receive support to deal with the identified stressors** | ‘Would you like to get support in dealing with any of the above difficulties and/or concerns? If so, please specify’ – open question added after focus group interviews as an attempt to maximize the clinical utility of HD-DT-C in renal care settings, following the adjustments made to the patient version of the tool (HD-DT) |
| **Decision on the name of the measure: Hemodialysis Distress Thermometer for Caregivers** | The abbreviation HD-DT-C was adopted for both English and Portuguese versions |

*Some of these changes followed those made in the patient version (HD-DT), as both measures were developed simultaneously and the same group of experts was involved in the evaluation of the HD-DT and HD-DT-C.
