# Supplementary material for: The Hemodialysis Distress Thermometer for Caregivers (HD-DT-C): development and testing of the psychometric properties of a new tool for screening psychological distress among family caregivers of adults on hemodialysis
Source: Qual Life Res. 2024 Mar 7;33(6):1513–26. doi: 10.1007/s11136-024-03627-x (PMC11116227; doi:10.1007/s11136-024-03627-x)
Supplement: Supplementary file 7 — Online Resource 7. Intra-rater values for the barometer and categorical items of the European-Portuguese version of the HD-DT-C during test-retest reliability. Supplementary file7 (DOCX 32 KB) [file 11136_2024_3627_MOESM7_ESM.docx]

**Online Resource 7.** Intra-rater values for the barometer and categorical items of the European-Portuguese version of the HD-DT-C during test-retest reliability (*n*=11 hemodialysis caregivers).

| **Items*** | ***ICC^1^*** | ***Kappa^2^*** | **95% *CI*** | | ***p*** |
| --- | --- | --- | --- | --- | --- |
|  |  |  | ***LB*** | ***UP*** |  |
| **DT barometer** | 0.991 | – | 0.959 | 0.998 | <0.001 |
| **Tiredness and/or fatigue** | – | 0.783 | 0.389 | 1.177 | 0.011 |
| **Sleep changes** | – | 0.800 | 0.435 | 1.165 | 0.010 |
| **Changes in sexual life/intimacy** | – | 1.000 | 1.000 | 1.000 | <0.001 |
| **Changes in physical ability (e.g., difficulty doing household chores, mobility, and moving around)** | – | 0.412 | -0.178 | 1.002 | 0.107 |
| **Changes in memory and/or concentration** | – | 1.000 | 1.000 | 1.000 | <0.001 |
| **Sadness and/or depression** | – | 0.800 | 0.435 | 1.165 | 0.010 |
| **Nervousness and/or anxiety** | – | 1.000 | 1.000 | 1.000 | <0.001 |
| **Feelings of grief and/or loss** | – | 1.000 | 1.000 | 1.000 | <0.001 |
| **Feelings of guilt (e.g., feeling I should do more for my family member on dialysis, feeling I should pay more attention to other family members)** | – | 0.800 | 0.435 | 1.165 | 0.010 |
| **Feeling overwhelmed by the responsibilities of caring for my family member** | – | 0.800 | 0.435 | 1.165 | 0.010 |
| **Concern about my family member's health** | – | 1.000 | 1.000 | 1.000 | <0.001 |
| **Fear that I will no longer be able to care for my family member** | – | 0.737 | 0.265 | 1.209 | 0.016 |
| **Concern about my family member's kidney transplant** | – | 0.737 | 0.265 | 1.209 | 0.016 |
| **Difficulty in dealing with my family member negative feelings (e.g., anger, sadness, hopelessness)** | – | 0.800 | 0.435 | 1.165 | 0.010 |
| **Not knowing how to support (e.g., encourage, reassure) my family member** | – | 0.800 | 0.435 | 1.165 | 0.010 |
| **Difficulty in accomplishing my goals and life projects** | – | 1.000 | 1.000 | 1.000 | <0.001 |
| **Caring for my family members affects my social and/or family life (e.g., less time/availability for vacation, leisure, work)** | – | 0.375 | -0.329 | 1.079 | 0.236 |
| **Lack of family support in the distribution of caregiving responsibilities** | – | 0.800 | 0.435 | 1.165 | 0.010 |
| **Problems with my family member's transportation (e.g., to dialysis and/or medical appointments)** | – | 0.615 | -0.046 | 1.276 | 0.035 |
| **Dealing with my family member's resistance to treatment (e.g., to dialysis sessions, fluid and/or dietary restrictions)** | – | 1.000 | 1.000 | 1.000 | <0.001 |
| **Difficulty denying liquids when my family member feels thirsty** | – | 1.000 | 1.000 | 1.000 | <0.001 |
| **Difficulty knowing how much liquids my family member can drink daily** | – | 1.000 | 1.000 | 1.000 | <0.001 |
| **Being creative with meals so that my family member does not lose his/her appetite** | – | 0.800 | 0.435 | 1.165 | 0.010 |
| **Difficulty denying certain foods to my family member** | – | 1.000 | 1.000 | 1.000 | <0.001 |
| **Knowing what is (or is not) recommended for my family member diet** | – | 0.783 | 0.389 | 1.177 | 0.011 |
| **Managing family meals and the care my family member needs with food** | – | 0.800 | 0.435 | 1.165 | 0.010 |
| **Taking care of my family member's vascular access (fistula or catheter)** | – | 1.000 | 1.000 | 1.000 | <0.001 |
| **Managing the different medications my family member takes** | – | 1.000 | 1.000 | 1.000 | <0.001 |
| **Lack of information about my family member's health status, treatments, and possible complications** | – | 1.000 | 1.000 | 1.000 | <0.001 |
| **Financial difficulties** | – | 1.000 | 1.000 | 1.000 | <0.001 |

*Notes*: ICC=Intraclass Correlation Coefficient; SE=Standard Error; LB=Lower Bound; UP=Upper Bound. Statistical significance was set at *p*<0.01.

* These items were translated for this table and do not correspond to the final American-English translation and cultural adaptation of the measure.

^1^ ICC values were interpreted as follow: <0.5 = poor, 0.5–0.75 = moderate, 0.75–0.9 = good, and > 0.90 = excellent reliability (Koo et al., 2016; Quinn et al., 2023).

^2^ Cohen's Kappa were interpreted as follow: ≤0 = no agreement, 0.01–0.20 = none to slight, 0.21–0.40 = fair, 0.41– 0.60 = moderate, 0.61–0.80 = substantial, and 0.81–1.00 = almost perfect agreement (Landis & Koch, 1977).

*References*

Koo, T. K., & Li, M. Y. (2016). A Guideline of Selecting and Reporting Intraclass Correlation Coefficients for Reliability Research. Journal of Chiropractic Medicine, 15(2), 155–163. <https://doi.org/10.1016/j.jcm.2016.02.012>

Landis, J. R., & Koch, G. G. (1977). The measurement of observer agreement for categorical data. Biometrics, 33(1), 159–174. Available in: https://dionysus.psych.wisc.edu/iaml/pdfs/landis_1977_kappa.pdf

Quinn, L., Tryposkiadis, K., Deeks, J., De Vet, H. C. W., Mallett, S., Mokkink, L. B., Takwoingi, Y., Taylor-Phillips, S., & Sitch, A. (2023). Interobserver variability studies in diagnostic imaging: a methodological systematic review. The British journal of radiology, 96(1148), 20220972. <https://doi.org/10.1259/bjr.20220972>
