# Supplementary material for: The Hemodialysis Distress Thermometer for Caregivers (HD-DT-C): development and testing of the psychometric properties of a new tool for screening psychological distress among family caregivers of adults on hemodialysis
Source: Qual Life Res. 2024 Mar 7;33(6):1513–26. doi: 10.1007/s11136-024-03627-x (PMC11116227; doi:10.1007/s11136-024-03627-x)
Supplement: Supplementary file 8 — Online Resource 8. Sensitivity and (1-)specificity for each cutoff point on the HD-DT-C barometer against the HADS-T cutoff score ≥15 for total distress. Supplementary file8 (DOCX 25 KB) [file 11136_2024_3627_MOESM8_ESM.docx]

**Online Resource 8.** Sensitivity and (1-)specificity for each cutoff point on the HD-DT-C barometer against the HADS-T cutoff score ≥15 for total distress.

| **Coordinates of the Curve** | | |
| --- | --- | --- |
| **Test Result Variable: HD-DT-F barometer** | | |
| **Positive if Greater Than or Equal To^a^** | **Sensitivity** | **1 - Specificity** |
| -1.00 | 1.000 | 1.000 |
| .50 | 1.000 | 0.969 |
| 1.50 | 1.000 | 0.797 |
| 2.50 | 1.000 | 0.578 |
| 3.50 | 0.976 | 0.313 |
| **4.50^b^** | **0.952** | **0.172** |
| **5.50^b^** | **0.905** | **0.094** |
| 6.50 | 0.786 | 0.047 |
| 7.50 | 0.571 | 0.031 |
| 8.50 | 0.262 | 0.000 |
| 9.50 | 0.024 | 0.000 |
| 11.00 | 0.000 | 0.000 |
| 1. The smallest cutoff value is the minimum observed test value minus 1. and the largest cutoff value is the maximum observed test value plus 1. All the other cutoff values are the averages of two consecutive ordered observed test values. 2. Optimum cutoff points, maximizing sensitivity and specificity. | | |
